# A novel approach for tetrahedral-element-based finite element simulations of anisotropic hyperelastic intervertebral disc behavior

**DOI:** 10.3389/fbioe.2022.1034441

**Published:** 2022-12-13

**Authors:** Marie-Rosa Fasser, Ramachandra Kuravi, Marian Bulla, Jess G. Snedeker, Mazda Farshad, Jonas Widmer

**Affiliations:** ^1^ Spine Biomechanics, Department of Orthopedic Surgery, Balgrist University Hospital, Zurich, Switzerland; ^2^ Institute for Biomechanics, ETH Zurich, Zurich, Switzerland; ^3^ Engineering Division, Lawrence Berkeley National Lab, Berkeley, CA, United States; ^4^ Altair Engineering GmbH, Cologne, Germany; ^5^ Department of Orthopedics, Balgrist University Hospital, Zurich, Switzerland

**Keywords:** intervertebral discs, spinal segments, microstructural modeling, FE-based models, explicit time-stepping, inverse FE method, tetrahedral elements

## Abstract

Intervertebral discs are microstructurally complex spinal tissues that add greatly to the flexibility and mechanical strength of the human spine. Attempting to provide an adjustable basis for capturing a wide range of mechanical characteristics and to better address known challenges of numerical modeling of the disc, we present a robust finite-element-based model formulation for spinal segments in a hyperelastic framework using tetrahedral elements. We evaluate the model stability and accuracy using numerical simulations, with particular attention to the degenerated intervertebral discs and their likely skewed and narrowed geometry. To this end, 1) annulus fibrosus is modeled as a fiber-reinforced Mooney-Rivlin type solid for numerical analysis. 2) An adaptive state-variable dependent explicit time step is proposed and utilized here as a computationally efficient alternative to theoretical estimates. 3) Tetrahedral-element-based FE models for spinal segments under various loading conditions are evaluated for their use in robust numerical simulations. For flexion, extension, lateral bending, and axial rotation load cases, numerical simulations reveal that a suitable framework based on tetrahedral elements can provide greater stability and flexibility concerning geometrical meshing over commonly employed hexahedral-element-based ones for representation and study of spinal segments in various stages of degeneration.

## 1 Introduction

The human spine is a complex arrangement of passive and active tissues (hard and soft) that provides postural control, flexibility of motion, and protects the spinal cord (Kibler et al., 2006; Agur and Dalley, 2009; Brittanica, 2014). Among these tissues, the hydrated soft collagenous intervertebral discs (IVD) separating adjacent vertebrae of the spine form major load-bearing elements (Widmer et al., 2020) that provide cushioning, regulate force distribution, and facilitate motion between spinal vertebrae (Humzah and Soames, 1988; Adams and Roughley, 2006; Roberts et al., 2006; Widmer et al., 2019). This multi-component complex structure in conjunction with the mechanical loads that it experiences during various physical activities (Schultz and Andersson, 1981; Schultz et al., 1982) results in complex internal load transfer mechanisms, which are expected to influence spinal pathologies such as back disorders and pain (Pearcy et al., 1984; Kumar, 1990; Dvořák et al., 1991; Manchikanti, 2000; Thiese et al., 2014) as well as secondary complications after surgical interventions such as adjacent segment disease (Bertagnoli, 2011), pseudoarthrosis (Steinmann and Herkowitz, 1992), and screw loosening (Bredow et al., 2016). In this context, finite element (FE) based models encompassing various spinal components have gained greater attention in recent decades to study spine biomechanics (Noailly et al., 2005; Schmidt et al., 2006; Campbell et al., 2016; Dreischarf et al., 2014; Zander et al., 2009; Schmidt et al., 2013; Jaramillo et al., 2015; Maquer et al., 2015; del Palomar et al., 2008; Ayturk et al., 2010; Zander et al., 2017) with increasing applications towards pre-clinical/surgical studies (Baroud et al., 2003; Rohlmann et al., 2007; Boccaccio et al., 2008; Talukdar et al., 2021), evaluating the influence of intervertebral disc degeneration (Schmidt et al., 2007b; Ayturk et al., 2012; Cegoñino et al., 2014), and towards subject-specific investigations (Widmer, 2020; Pickering et al., 2021). These computationally powerful tools are particularly effective in combining hierarchic intricacies of complex spinal systems with material and geometrical non-linearities and a wide range of loading scenarios (Schmidt et al., 2013). However, the performance of these FE models (i.e., accuracy, computational efficiency, and robustness) is greatly influenced by 1) component-specific material models and the corresponding model parameters (in particular for soft tissues like the IVD), 2) accuracy and discretization of the three-dimensional geometry, and 3) numerical solution techniques employed.

Due to complex inner microstructure and internal hierarchy (see Section 2.1), the IVD exhibits highly non-linear behavior (Markolf and Morris, 1974; Goel et al., 1995; Ebara et al., 1996; Eberlein et al., 2001; Wagner and Lotz, 2004). Various mathematical models were proposed in the literature, with different mathematical treatments for the nucleus pulposus (NP) and annulus fibrosus (AF) components of the IVD. Example modeling approaches are linearised-elasticity-based models (Harkness, 1961; Haut and Little, 1972; Ueno and Liu, 1987; Smit et al., 1997; Baroud et al., 2003; Polikeit et al., 2003), non-linear composite models with a one-dimensional description of collagen fibers (Schmidt et al., 2006; 2007b; Rohlmann et al., 2007; Zander et al., 2009; Dreischarf et al., 2014; Pickering et al., 2021), microstructure-based continuum non-linear models (Eberlein et al., 2001, 2004; Ayturk et al., 2012, 2010; Jaramillo et al., 2015), and micromechanical models (Ghezelbash et al., 2021). Continuum material models are often employed to model the IVD as they are particularly advantageous to represent its material non-linearity while implicitly incorporating its anisotropy to the desired degree of accuracy (Holzapfel et al., 2015, 2005) besides offering relative ease for FE implementation. However, ambiguity with the corresponding material parameters prevails because various parameters suggested in the literature (Moramarco et al., 2010; del Palomar et al., 2008; Jaramillo et al., 2015; Wagner and Lotz, 2004; Eberlein et al., 2001, 2004) were each attuned to a specific set of experiments that were largely uni-directional in nature (Markolf and Morris, 1974; Goel et al., 1995; Ebara et al., 1996; Eberlein et al., 2001; Wagner and Lotz, 2004). This suggests a need for robust calibration using multi-directional experimental data to increase the model’s predictive abilities.

Various image-processing-based methods are in use to generate FE models for spinal segments. These approaches use computed tomography (CT) images of vertebrae (Moramarco et al., 2010; del Palomar et al., 2008; Rohlmann et al., 2007; Eberlein et al., 2004; Schmidt et al., 2006; Pickering et al., 2021; Jaramillo et al., 2015) and magnetic resonance imaging (MRI) scans of IVDs (Maquer et al., 2015, 2014), including those based on automatic segmentation (Caprara et al., 2021). Hexahedral elements (HE) often feature in the subsequent discretization of the resulting geometry, in particular of healthy IVDs (Eberlein et al., 2001, 2004; Jaramillo et al., 2015; Moramarco et al., 2010; Rohlmann et al., 2007; Pickering et al., 2021; Baroud et al., 2003; Schmidt et al., 2006, 2007b,a; Zander et al., 2009, 2017; Cegoñino et al., 2014). This is because, 1) HE mesh regular geometries easily and with fewer elements, while offering high solution accuracy (Benzley et al., 1995), 2) they can be arranged parallel to the IVD circumference with one of the local coordinate axes being tangent to it and therefore simplifying the identification of local collagen fiber directions, and 3) HE in the form of hourglass controlled reduced integration overcome the volumetric locking problem in incompressible soft materials (Bathe, 2006; Hughes, 2012).

However, degeneration-induced changes significantly affect the geometry of IVDs. Specifically, there can be a considerable drop in height, an accumulation of tears in the annulus region, and endplate effects (Adams and Roughley, 2006; Widmer, 2020). Furthermore, olisthesis or dorsal disc narrowing can result in strongly skewed or wedged-shaped IVDs. Such complex geometries are extremely difficult to reproduce with (homogeneously sized) HE and if meshed inaptly this can cause numerical instabilities. This mandates using computationally expensive and laborious high-quality HE meshes. In this regard, tetrahedral elements (TE) are commonly employed in literature to discretize complex geometries with relative ease due to their superior flexibility and adaptability (Bathe, 2006; Hughes, 2012; Schneider et al., 2019). Furthermore, volumetric locking can be addressed with a suitable choice of integration schemes (e.g., reduced, selective reduced) (Bathe, 2006; Hughes, 2012). Refined approaches in the context of volumetric locking issues with TE elements have also been explored (Joldes et al., 2009; Pagani et al., 2014). Finally, while implicit finite element analysis is generally faster for linear problems, explicit numerical solution techniques are often selected over implicit methods in addressing quasi-static problems (with negligible inertial effects) in FE methods (FEM) because 1) no iterations are required to evaluate solution variables, 2) evaluation of computationally expensive inverse stiffness is not required, 3) material and geometric non-linearities, as well as contacts, are handled better, 4) high levels of efficiency are possible with parallelization for analyses solved using multiple processors. Yet, an optimal choice of the time step is paramount to ensure the desired accuracy of numerical solutions while maintaining computational efficiency. While this time step is deformation-dependent (Ogden, 1997), it is traditionally prescribed as a suitably small constant (dependent on the material parameters) in typical explicit FE solvers such as Ansys LS-DYNA and Radioss for ease of implementation in a range of problems in mechanics (Freed et al., 2005; Wu et al., 2020). However, in highly non-linear anisotropic hyperelastic materials like IVD tissue, this time step can be noticeably influenced by the state of deformation and local material symmetry, suggesting a re-evaluation of the traditional approach.

This project focuses on establishing a novel and robust FE-based model formulation for spinal segments using TE in a hyperelastic framework. To this end, 1) a microstructure-based continuum anisotropic material formulation is utilized for the simulation of AF behavior in an explicit-time-integration-based numerical framework. 2) An adaptive time-stepping approach is proposed as a computationally efficient approximation to a refined deformation-dependent alternative (Ogden, 1997). Finally, 3) the performance of linear HE and TE is evaluated in terms of their accuracy and stability during flexion, extension, lateral bending, and axial rotation, using spinal FE models with non-degenerated, moderately, and severely degenerated IVDs. Also, a material parameter set is estimated using experimental data of spinal segments during the above load cases (Widmer et al., 2020) and an inverse FE-based approach.

## 2 Methods

### 2.1 Continuum material formulations for the IVD

The internal microstructure of the IVD consists of an inner NP enclosed by an outer AF and cartilage endplates anchoring the IVD to the vertebrae. Both AF and NP are predominantly filled with water (65%–90% in AF (Marcolongo et al., 2017) and 70%–88% in NP (Humzah and Soames, 1988; Marcolongo et al., 2017)) and a proteoglycan matrix into which collagen fiber bundles are embedded (Hashizume, 1980; Inoue, 1981; Cassidy et al., 1989). Collagen fiber bundles in AF are arranged into several layers of concentric lamellae with alternating orientations ranging between 25° and 45° (Cassidy et al., 1989; Adams and Roughley, 2006; Ambard and Cherblanc, 2009; Malandrino et al., 2013) about the transverse plane, while in NP they are randomly oriented in a homogeneous matrix (Hashizume, 1980; Inoue, 1981). The arrangement of the collagen fibers in AF is theorized to resist the high tensile hoop loads resulting from the hydrostatic pressure transferred from the nucleus pulposus during spinal compression by helping to absorb and redistribute stresses. The complex internal hierarchy and microstructure can be linked to the experimentally determined non-linear mechanical behavior observed both at the component and the tissue level (Markolf and Morris, 1974; Goel et al., 1995; Ebara et al., 1996; Eberlein et al., 2001; Wagner and Lotz, 2004; Holzapfel et al., 2005). In this study, IVD is modeled as a multi-component system with individual constitutive material models for AF and NP, to incorporate its complex three-dimensional microstructure. To this end, both AF and NP are modeled as hyperelastic solid bodies utilizing an invariant-based formulation (Spencer, 1984; Holzapfel, 2002) wherein pressure and displacement are decoupled for numerical ease (Flory, 1961; Ogden, 1978; Simo and Hughes, 2006).

#### 2.1.1 Kinematics and preliminaries

In line with the standard notation in continuum mechanics, let the configuration of a body B in the reference and current (at time *t*) states be denoted by $\varkappa_{R}\left(B\right)$ and $\varkappa_{t}\left(B\right)$ , respectively. Each material point of B corresponds with the positions $X\in \varkappa_{R}\left(B\right)$ and $x\in \varkappa_{t}\left(B\right)$ , which are linked through the mapping $x=\chi_{\varkappa_{R}}\left(X,t\right)$ . The deformation gradient **F**(***X***, *t*), its determinant *J*(***X***, *t*), and the right Cauchy-Green tensor **C**(***X***, *t*) are defined through

$$F=\text{Grad}\chi_{\varkappa_{R}},\;\;J=\text{det}F>0,\;\;C=F^{T}F,$$ (1)

where the dependence on location and time is understood. Densities in the current (*ρ*) and reference states (*ρ*_0_) are related through *ρ* = *Jρ*_0_. The strain energy density function (SEDF) Ψ of each hyperelastic material component depends on **F** through the right Cauchy-Green tensor **C** = **F** ^T^ **F** which, in the decoupled form, becomes (Holzapfel et al., 2000)

$$\Psi =\bar{\Psi }\left(\bar{C}\right)+\tilde{\Psi }\left(J\right),$$ (2)

where $\bar{C}={\bar{F}}^{T}\bar{F}$ and $\bar{F}=J^{-1/3}F$ . $\bar{\Psi }$ and $\tilde{\Psi }$ represent the distortional and dilatational isotropic strain energies, respectively. Furthermore, for an anisotropic hyperelastic material reinforced with *n* families of fibers whose directions are specified by unit vectors ***M***_*i*_, *i* = 1, 2, … , *n*, the SEDF can be given as (Holzapfel and Weizsäcker, 1998; Holzapfel et al., 2000; Truesdell and Noll, 2004)

$$\Psi =\bar{\Psi }\left(\bar{C}\right)+\tilde{\Psi }\left(J\right)+\hat{\Psi }\left(\bar{C},M_{i}\right),$$ (3)

where **M**_*i*_≔ ***M***_*i*_ ⊗ ***M***_*i*_ and $\hat{\Psi }$ represents the distortional anisotropic strain energy. The second Piola-Kirchhoff stress (**S**) and the Cauchy stress (***σ***) are then obtained as

$$S=2\frac{\partial \Psi }{\partial C},\;\sigma =J^{-1}{FSF}^{T}$$ (4)

Finally, Cartesian components of the material $\left(C_{ijkl}\right)$ and spatial (*c*_*ijkl*_) elasticity tensors are obtained as (Ogden, 1997; Holzapfel, 2002)

$$C_{ijkl}=2\frac{\partial S_{ij}}{\partial C_{kl}},\;c_{ijkl}=J^{-1}F_{iP}F_{jQ}F_{kR}F_{lS}C_{PQRS}$$ (5)

#### 2.1.2 Nucleus pulposus

The behavior of the NP is modeled using a compressible Mooney-Rivlin type formulation given as (Holzapfel, 2002)

$$\Psi_{NP}=b_{10}\left({\bar{I}}_{1}-3\right)+b_{01}\left({\bar{I}}_{2}-3\right)+\frac{k_{NP}}{2}{\left(J-1\right)}^{2},$$ (6)

where ${\bar{I}}_{1}≔tr\bar{C}$ and ${\bar{I}}_{2}≔tr{\bar{C}}^{-1}$ are kinematic invariants, *b*_10_ and *b*_01_ are positive material constants with the units of stress and *k*_NP_ is the bulk modulus. Herein, it is noted that the NP is modeled as an isotropic material despite the presence of collagen fibers because of their random and homogeneous distribution in the matrix [see also Schmidt et al. (2006, 2007a,b); Ayturk et al. (2012)] The material model parameters for NP adopted from Schmidt et al. (2006, 2007a) and Ayturk et al. (2012) are listed in Table 1.

**TABLE 1:** Material model parameters of NP and AF, where *k*_NP_ and *k*_AF_ are obtained from their respective Poisson’s ratios of 0.495 (Ayturk et al., 2012) and 0.45 (Goel et al., 1995).

Material	Parameter	Value
Nucleus pulposus	*b*_10_ (kPa)	0.12
	*b*_01_ (kPa)	0.03
	*k*_NP_ (kPa)	29.9
Annulus fibrosus	*c*_10_ (kPa)	0.18
	*c*_01_ (kPa)	0.045
	*a*_1_ (kPa)	2
	*a*_2_	100
	*k*_AF_ (kPa)	4.35

#### 2.1.3 Annulus fibrosus

To model the anisotropic mechanical response of the AF, a modified Mooney-Rivlin type formulation incorporating contributions from two collagen fiber families ***M***_1_ and ***M***_2_ is utilized based on (Eberlein et al., 2001; Eberlein et al., 2004; Moramarco et al., 2010). Therefore, $\Psi \left(\bar{C},J,M_{1},M_{2}\right)$ from Eq. 3 becomes

$$\Psi_{AF}=c_{10}\left({\bar{I}}_{1}-3\right)+c_{01}\left({\bar{I}}_{2}-3\right)+\frac{k_{AF}}{2}{\left(J-1\right)}^{2}+\overset{2}{\underset{i=1}{\sum }}\frac{a_{1}}{a_{2}}\left\{\text{exp}\left(a_{2}{\left\langle {\bar{I}}_{M}^{\left(i\right)}-1\right\rangle }^{2}\right)-1\right\},$$ (7)

where the invariant ${\bar{I}}_{M}^{\left(i\right)}≔tr\left(\bar{C}M_{i}\right)$ measures the squared stretch of fibres along ***M***_*i*_. *c*_10_, *c*_01_ and *a*_1_ are material parameters with units of stress, *a*_2_ is a dimensionless constant, *k*_AF_ is the bulk modulus, and ⟨*x*⟩ = (|*x*| + *x*)/2. It is noted that only tensile stretch of collagen fibers is considered due to their crimped structure (Cassidy et al., 1989). In the current study, a homogeneous distribution of collagen fiber bundles is assumed, despite their alternating orientation in the lamellae, exploiting the periodic and concentric nature of the lamellae (Eberlein et al., 2001, 2004; Moramarco et al., 2010). Furthermore, ***M***_1_ and ***M***_2_ are assumed to be at an average orientation of ±30° about the transverse plane (Goel et al., 1995; Eberlein et al., 2001; Urban and Roberts, 2003; Moramarco et al., 2010). While the orthotropic material symmetry of the AF described by ***M***_1_ and ***M***_2_ is evident, the same can be described by the directions ***M***_1_ + ***M***_2_ and ***M***_1_ − ***M***_2_ with relative ease^1^. The material model parameters of the AF used for the IVD FE model testing (Section 2.3.1) are listed in Table 1 and are in accordance with previous literature findings (Schmidt et al., 2006; 2007b; Ayturk et al., 2012).

### 2.2 Adaptive time step

Invoking the theory of infinitesimal waves and vibrations in unbounded materials in the context of finite deformations (Ogden, 1997), the acoustic tensor $\tilde{Q}\left(n\right)$ and the wave speed *v* of a plane given by ***v*** = ***m*** *f* (***n*** ⋅ ***x*** − *vt*) are related through

$$\rho v^{2}=\left[\tilde{Q}\left(n\right)m\right]\cdot m,\;{\tilde{Q}}_{jl}=A_{ijkl}n_{i}n_{k},\;A_{ijkl}=c_{ijkl}+\sigma_{ik}\delta_{jl},$$ (8)

where unit vectors ***n*** and ***m*** denote the direction of wave propagation and polarisation of the wave, respectively. {*n*_1_, *n*_2_, *n*_3_}, {*m*_1_, *m*_2_, *m*_3_}, and *δ*_*ij*_, respectively, represent the Cartesian components of ***m***, ***n***, and the identity tensor. *A*_*ijkl*_ is stiffness tensor. For longitudinal (P-) waves defined through ***n*** = ***m***, Eq. 8 simplifies to

$$v=\sqrt{\frac{J}{\rho_{0}}\left(c_{ijkl}+\sigma_{ik}\delta_{jl}\right)n_{i}n_{j}n_{k}n_{l}}$$ (9)

Noteworthy, the local wave speed in anisotropic materials is strongly influenced by the local fiber directions (Eqs 5, 7, 8). To this end, let *n*_1_ = cos *ϕ* sin *θ*, *n*_2_ = sin *ϕ* sin *θ*, and *n*_3_ = cos *θ* without loss of generality and with *ϕ* ∈ [0, *π*) and *θ* ∈ [0, *π*). Then, the maximum local wave speed (*v*_max_) and the corresponding direction of propagation for a given state of deformation (**F**, ***σ***) can be deduced by maximizing a suitable objective function $\hat{v}\left(\phi ,\theta \right)$ ^2^ i.e.,

$$v_{\max}=\max\;\hat{v}\left(\phi ,\theta \right)$$ (10)

While the estimation of wave speed using Eq. 10 is essential for numerical simulations using the explicit FEM (see Section 2.3), it also increases the overall computational effort. Therefore, in this study *v*_max_ is approximated as

$$v_{\max}=\max\;\tilde{v}\left(\tilde{M}\right),\;\tilde{M}\in M≔\left\{M_{1},M_{2},M_{1}+M_{2},M_{1}-M_{2},M_{1}\times M_{2}\right\}$$ (11)

i.e, as the maximum of the wave speeds along the set of directions M , by exploiting (i) the local orthotropy of the AF and (ii) substantially higher collagen fiber stiffness over the matrix as a rectification over the commonly used constant dilatational wave speed approach (Freed et al., 2005; Wu et al., 2020). We discuss this in Section 3, wherein the choice of M is compared against the theoretically estimated propagation direction (Eq. 10).

### 2.3 FE modeling

Numerical simulations were performed on three different FE models of lumbar spine segments representing various stages of IVD degeneration (Supplementary Figure S1).

**FIGURE 1:**
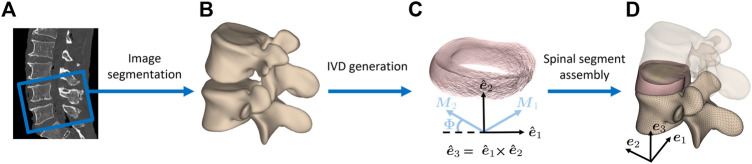
Finite element model generation of lumbar spine segments. **(A)** CT images of human cadaveric spines were used to obtain **(B)** vertebral 3D models through segmentation. **(C)** The endplates of the resulting vertebral surfaces were used to create the intervertebral disc geometry in between. Local collagen fiber directions ***M***_1_ and ***M***_2_ of the AF are displayed. {***e***_1_, ***e***_2_, ***e***_3_} and $\left\{{\hat{e}}_{1},{\hat{e}}_{2},{\hat{e}}_{3}\right\}$ are respectively the global and local orthonormal bases. **(D)** Finally, the components of the spinal segment were assembled into one FE model composed of bony and soft tissue (translucent upper vertebra for illustration purposes only).

The 3D geometrical mesh information of individual bony structures (i.e., cranial and caudal vertebra of each segment) was obtained from manual segmentation of CT images (Philips Brilliance 64, Philips Healthcare, Cleveland, OH, United States) using the 3D Slicer (V4.8.1) (3D Slicer, 2021; Fedorov et al., 2012) software (Figure 1). Statistical shape models were transformed onto this outcome by utilizing specific landmarks and invoking a non-rigid registration approach (Caprara et al., 2021). This information was then utilized in conjunction with custom-made scripts in MATLAB^®^ (The MathWorks Inc., Natick, MA, United States) to generate the 3D geometry of the enclosed IVD. For this purpose, the surface geometry of the cranial vertebra’s lower endplate and the caudal vertebra’s upper endplate were used. The circumferential profile of the IVD was shaped by a modest radial translation of the corresponding nodes to shape a gentle outward curvature. The location, shape, and size of the NP near the center of the IVD were defined based on anatomical studies (Pooni et al., 1986; O’Connell et al., 2007).

**FIGURE 2:**
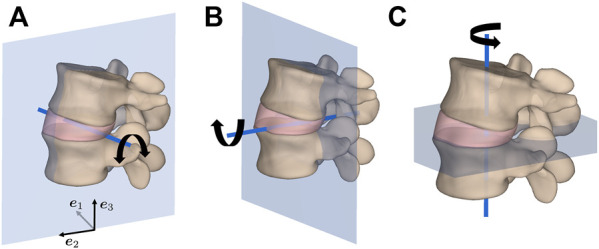
Load cases of **(A)** flexion/extension in the sagittal plane, **(B)** lateral bending in the frontal plane, and **(C)** axial rotation in the transverse plane for an applied moment of 5 Nm about the illustrated axis of rotation through the geometric center of the IVD.

In this study, the load cases of 1) flexion, 2) extension, 3) lateral bending, and 4) axial rotation in the three FE models of spinal segments were considered, as illustrated in Figure 2. To this end, the upper cranial vertebra was subjected to a moment of 5 Nm about an axis through the centroid of the IVD and normal to the sagittal plane for (1) and (2), frontal plane for (3), and transverse plane for (4). The IVD was rigidly connected to both vertebrae at their respective interfacing surfaces through a nodal tie constraint and the lower caudal vertebra was allowed only to rigidly translate in the moment plane. The purpose of this last constraint was to closely replicate the conditions of previously conducted biomechanical experiments (Widmer et al., 2020; Cornaz et al., 2021).

The critical time step for Radioss explicit FE solver is estimated as

$$\Delta t_{crit}=F\frac{l_{c}}{v_{max}},$$ (12)

Invoking Eq. 11 where *l*_*c*_ and F=0.9 are the characteristic element length deduced from the FE mesh and a multiplicative adjustment factor, respectively. The choice of 0.9 for this multiplication factor was based on a rational compromise between maintaining a near-optimal computational effort and providing a reasonable buffer below the estimated minimum time step.

#### 2.3.1 IVD degeneration and solid element type

We tested the described approach for IVD modeling by generating three different spinal segment geometries corresponding to L4-L5, L1-L2, and L2-L3 encompassing non-degenerated, moderately, and severely degenerated IVDs, respectively. Numerical simulations were performed on these three different FE models of lumbar spine segments with the IVD geometry being volumetrically discretized once with linear HE and once with linear TE. While the volumetric discretization of the IVD using hexahedral elements was performed in MATLAB, Hypermesh [HyperMesh, version 2017.2, Altair Engineering Inc., Troy, United States (HyperMesh, 2017)] was utilized for the tetrahedral-element-based discretization of the same.

All mechanical analyses were performed through a FE simulation of the corresponding boundary value problems with the explicit FE solver Radioss (2019). The load cases considered in this regard were implemented on a domain comprising the two vertebrae encompassing the IVD. Due to the considerable difference between the mechanical stiffness of the spinal vertebrae and the IVD (Lu et al., 1996; Baroud et al., 2003; Schmidt et al., 2006; Rohlmann et al., 2007), the former are modeled as rigid bodies. For both of the considered solid element types, a built-in Mooney-Rivlin-type material model in Radioss was used for NP, while the material model for AF followed the description of Sections 2.1 and 2.2 and was implemented through a user-defined material subroutine. Herein, the local collagen fiber directions (***M***_1_ and ***M***_2_) in the local orthonormal basis $\left\{{\hat{e}}_{1},{\hat{e}}_{2},{\hat{e}}_{3}\right\}$ are defined as

$$M_{1}=\left[\;\cos\Phi ,\sin\Phi ,0\;\right],\;M_{2}=\left[\;\cos\Phi ,-\sin\Phi ,0\;\right],$$ (13)

where Φ represents the orientation of the fibers about the local transverse plane spanned by ${\hat{e}}_{1}$ and ${\hat{e}}_{3}$ . These vectors are then related to the element-specific coordinate system $\left\{{\bar{e}}_{1},{\bar{e}}_{2},{\bar{e}}_{3}\right\}$ through an orthogonal transformation $Q_{R}:\left\{{\hat{e}}_{1},{\hat{e}}_{2},{\hat{e}}_{3}\right\}\to \left\{{\bar{e}}_{1},{\bar{e}}_{2},{\bar{e}}_{3}\right\}$ . The element-specific coordinate system is defined for the solver and based on the element edges and the sequence of the corresponding nodes (Radioss, 2019). The Euler angles of **Q**_*R*_ (Varshalovich et al., 1988) were determined in a pre-processing step performed in MATLAB with custom scripts using elemental edge (for HE) and orientation (for HE and TE) information and were provided as an initial input to the FE solver. Noteworthy, elemental collagen fiber orientations were determined by considering their tangency to the IVD outer circumference, a consequence of the concentric alignment of the lamellae (Holzapfel et al., 2005). Supplementary Table S1 summarizes the details of the FE discretization for all the models utilized in this study. Herein, it is noted that the vertebrae are discretized using shell-type elements with a thickness of 0.05 mm to associate non-zero inertia. Furthermore, a homogeneous FE mesh was considered to facilitate ease of meshing. Mesh dependency for the case of flexion is explored in Supplementary Figure S2 of the supplementary material wherein the mesh size was varied over 2.2 mm for coarse and 1.0 mm for refined meshes. Herein, it is noted that at 1.4 mm nominal size the maximum discretization error in relevant parameters was under 3% compared to the refined mesh. Therefore, 1.4 mm was chosen as a reasonable compromise between accuracy and computational efficiency (Supplementary Figure S2).

To compare the results of maximum longitudinal wave speeds determined by Eqs 10, 11 with each other, numerical simulations were first performed on the FE model of a spinal segment with non-degenerated IVD with TE and using a small value of Δ*t*_crit_ ^3^. Thereafter, the desired elemental state variables ({**F**, ***σ***}^e^) at the peak loading state were extracted. Finally, these output variables were used in conjunction with Eqs 10, 11 to estimate the corresponding elemental wave speeds. To this end, all four load cases were considered and material parameters based on the results of Eberlein et al. (2001); Ayturk et al. (2012); Goel et al. (1993, 1995) were assumed for AF.

Numerical simulations were performed on the FE models considering two different mesh element types, i.e., linear hexahedral and linear tetrahedral, and the corresponding results were compared with each other in terms of the distributions of pressure *p*, isotropic energy density $\bar{\Psi }$ , and anisotropic energy density $\hat{\Psi }$ . *p* is defined as (Rajagopal, 2015)

$$p=-\frac{tr\left(\sigma \right)}{3}$$ (14)

Further, simulation results (i.e., stability and load-displacement) obtained using HE and TE types were compared for various degeneration states (Supplementary Figure S1A). Simulations providing results for the prescribed load without extreme deformation and buckling of single elements (causing the simulation to stop) were considered to be stable.

#### 2.3.2 AF material parameters and inverse FEM

A set of material parameters of AF were estimated using inverse FEM and the calculations were performed on the spinal segment model with non-degenerated IVD meshed with TE elements (Supplementary Table S1). The experimental data for the four load cases were obtained from Widmer et al. (2020) and the average results for 31 non-degenerated spine segments were considered to be the experimental reference.

**FIGURE 3:**
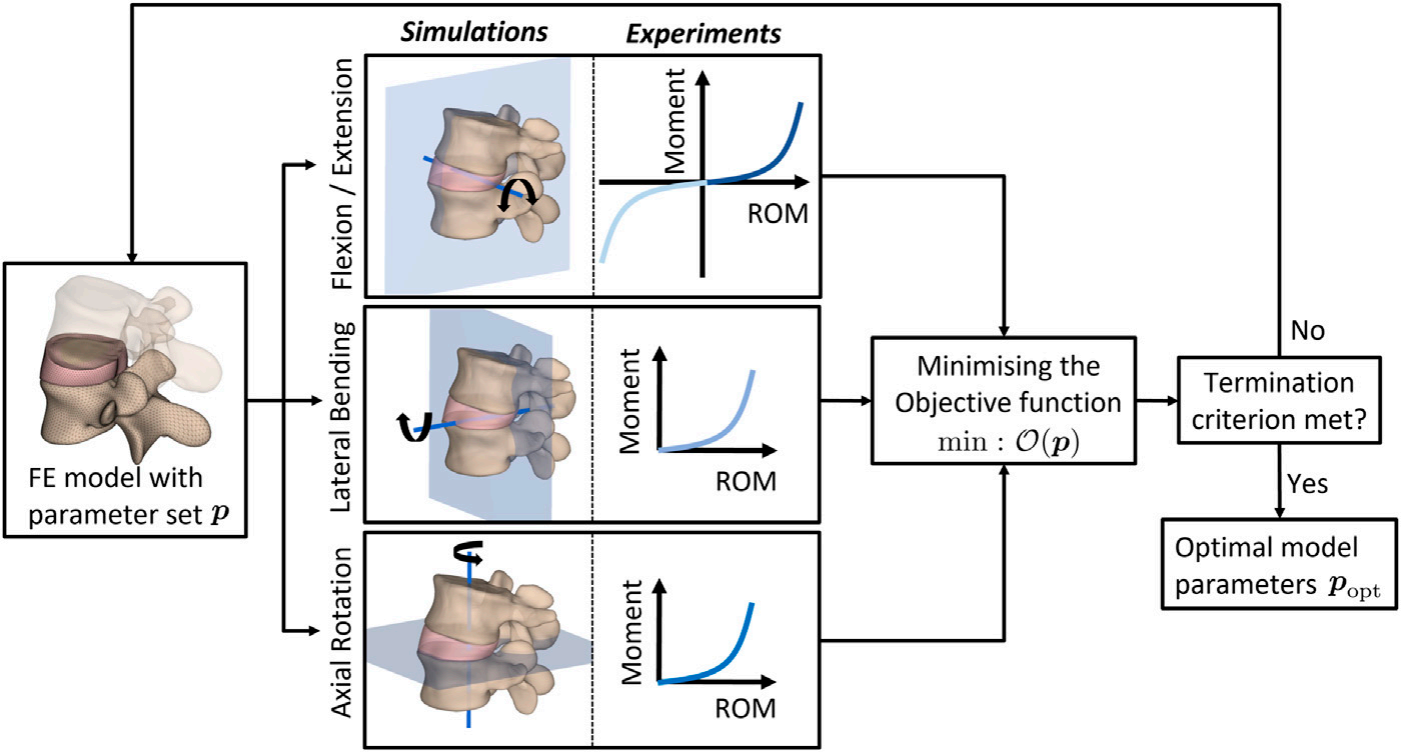
Flowchart of the MATLAB-driven inverse-FEM-based optimization algorithm. The material properties characterizing the AF behavior are optimized towards the best agreement between experimental measurements and results of finite element simulations emulating the experiments. ROM: range of motion.

Briefly, the desired material parameter set (***p***_opt_) of the AF is determined using an optimization algorithm implemented in MATLAB that iteratively minimizes the difference between numerically simulated responses from FE analyses performed in Radioss and the corresponding experimental data (see e.g., Ahn and Kim (2010); Kuravi et al. (2021); Böl et al. (2013)). To this end, the sequential quadratic programming algorithm (Wright and Nocedal, 2006) implemented in MATLAB’s *fmincon* function was invoked to minimize the objective function given as

$$O\left(p\right)=\sqrt{\frac{1}{n}\overset{n}{\underset{i=1}{\sum }}{\left(\frac{\Theta_{i}^{\exp}-\Theta_{i}^{sim}\left(p\right)}{\Theta_{n}^{\exp}}\right)}^{2}},$$ (15)

where $\Theta_{i}^{\exp}$ and $\Theta_{i}^{sim}\left(p\right)$ , respectively, denote the experimental and simulated range of motion for a given parameter set ***p*** while *n* denoted the number of load increments. Figure 3 summarises the optimization algorithm.

## 3 Results

In the first part of the results section, the outcome of time step estimation using a deformation-dependent and computationally efficient method are reported (Section 3.1). Thereafter, numerical simulation results using HE and TE are presented and compared with each other for the considered four load cases and for models with varying levels of degeneration (Section 3.2). Finally, material parameters based on experimental results obtained for non-degenerated IVDs through inverse FEM are reported (Section 3.3).

### 3.1 Time step estimation for explicit FEM

**FIGURE 4:**
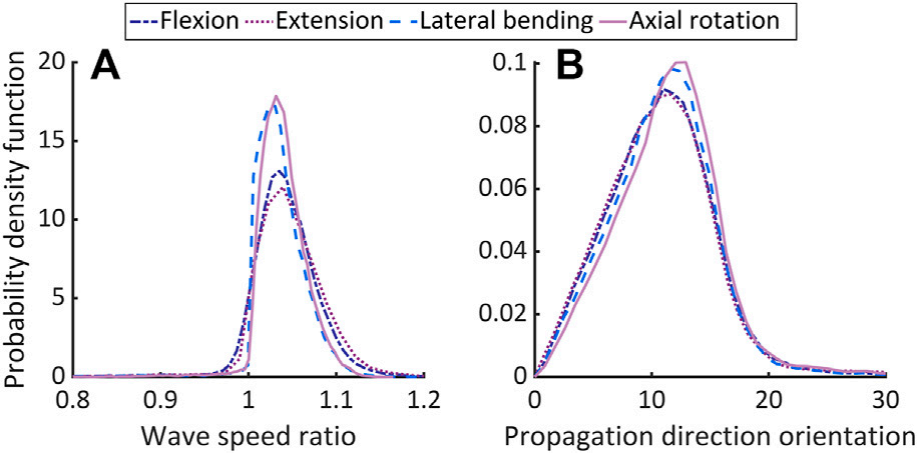
Comparing the theoretically predicted and approximated longitudinal wave speeds in non-degenerated AF. Plot **(A)** shows the ratio of wave speeds from Eq. 10 and the maximum from the directions in M and plot **(B)** shows the angular separation between the corresponding propagation directions, both expressed as probability density functions. $\bar{\mu }$ and SD, respectively, range in (1.033–1.038), (0.0281–0.0510) for **(A)**, and (10.56–11.12), (4.75–6.25) for **(B)**.

Figures 4A,B, respectively, depict probability density functions of the ratio of wave speeds and the corresponding angular separation estimated from Eqs 10, 11 for all elements of AF (Supplementary Table S1). The corresponding mean $\left(\bar{\mu }\right)$ and standard deviation (SD) values are given in Table 2.

**TABLE 2:** Comparing theoretical and approximated (P-) wave speed parameters.

Load case	Wave speed ratio ( $\bar{\mu }$ , SD)	Angular separation ( $\bar{\mu }$ , SD)
Flexion	(1.038, 0.0404)	(10.56, 5.22)
Extension	(1.033, 0.0510)	(11.00, 6.25)
Lateral bending	(1.033, 0.0385)	(10.93, 5.256)
Axial rotation	(1.0368, 0.0281)	(11.12, 4.75)

### 3.2 Spinal model predictions for HE and TE

**FIGURE 5:**
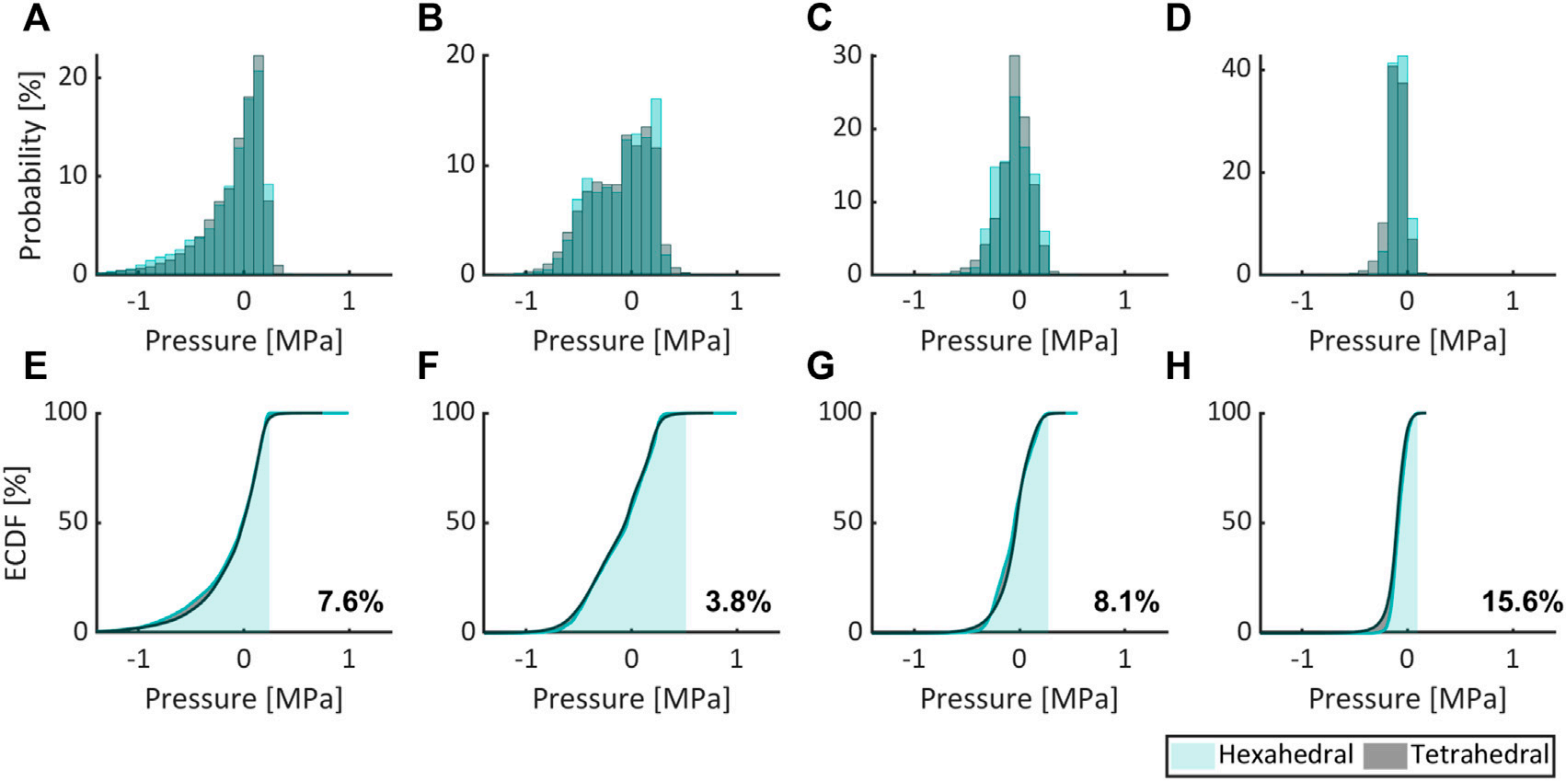
Comparing the mechanical pressure expressed as probability (normalized histogram) and ECDF for flexion **(A,E)**, extension **(B,F)**, lateral bending **(C,G)**, and axial rotation **(D,H)** for HE and TE types.

**FIGURE 6:**
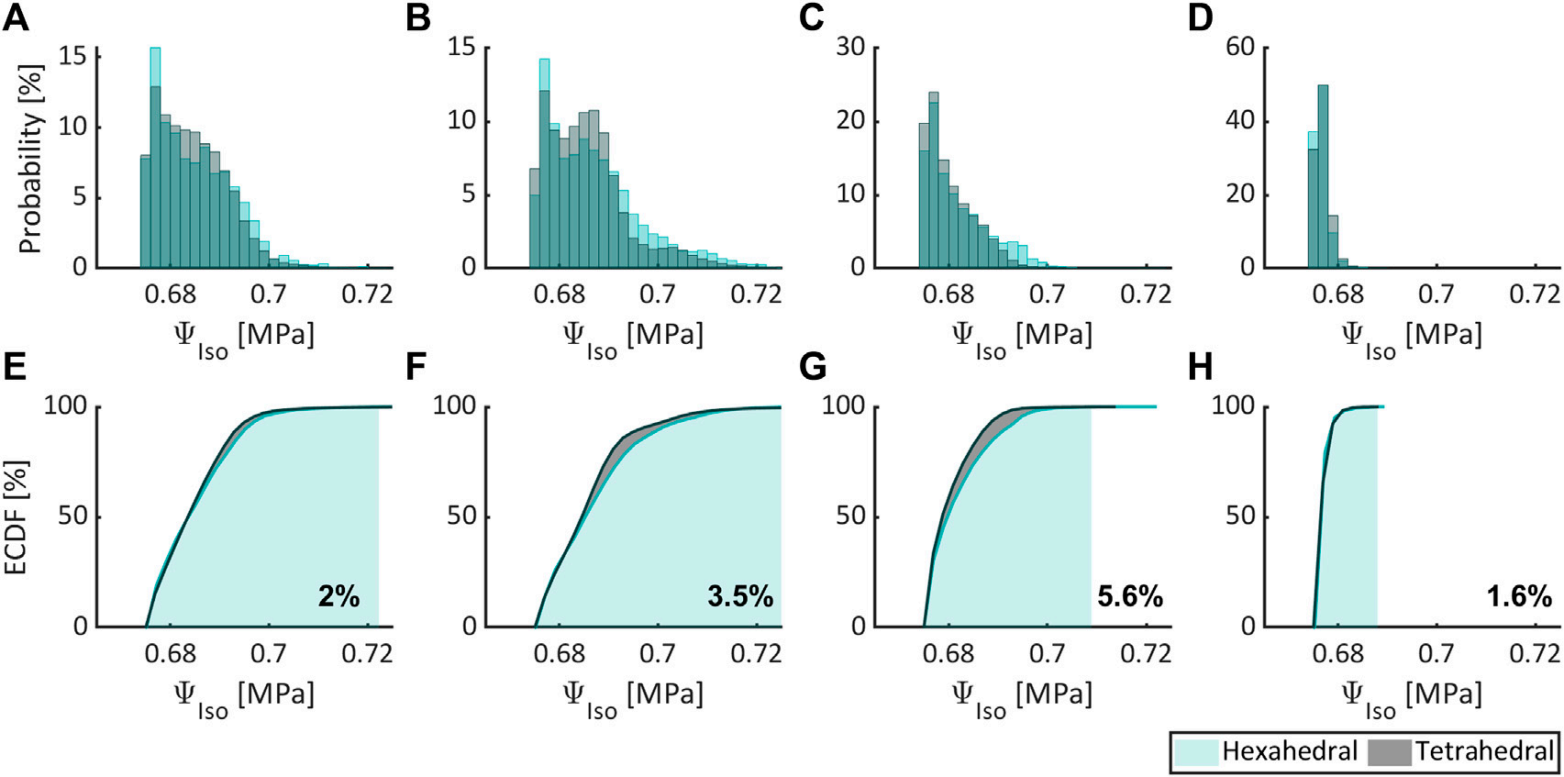
Comparing the isotropic contribution to the SEDF expressed as probability (normalized histogram) and ECDF for flexion **(A,E)**, extension **(B,F)**, lateral bending **(C,G)**, and axial rotation **(D,H)** for HE and TE types.

**FIGURE 7:**
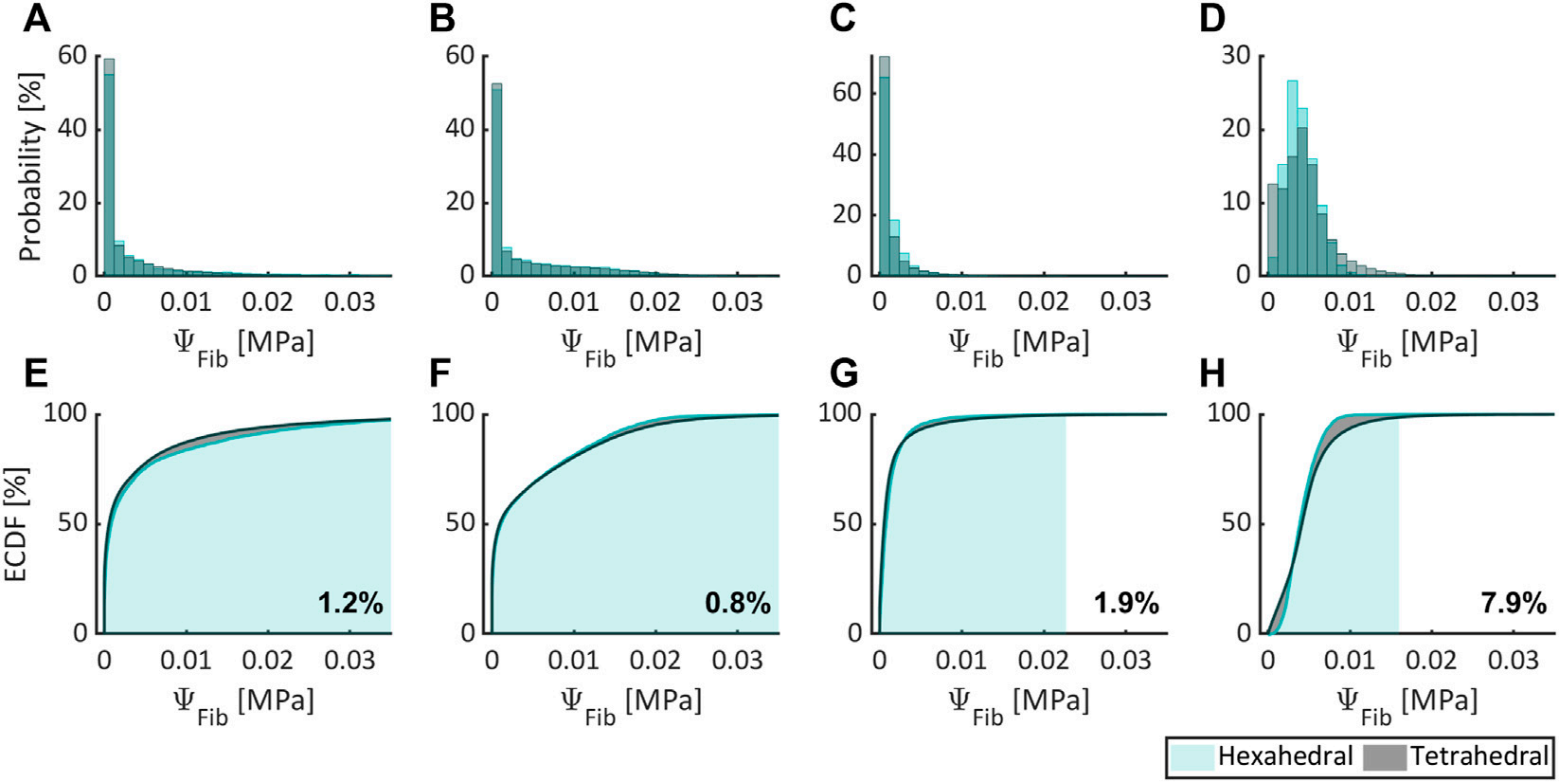
Comparing the anisotropic contribution to the SEDF expressed as probability (normalized histogram) and ECDF for flexion **(A,E)**, extension **(B,F)**, lateral bending **(C,G)**, and axial rotation **(D,H)** for HE and TE types.

Figure 5–7, respectively, compare the state variables of mechanical pressure, isotropic (distortional), and anisotropic contributions to the SEDF of the numerical simulations obtained from a non-degenerated IVD meshed either with HE or with TE. Normalized histograms and empirical cumulative distribution functions (ECDF) were used to illustrate the distribution of the values in the elements. ECDF is a continuous function depicting the number of observations in percentile i.e., the percentage of observations that are less or equal to the value at a given point on X-axis.

Only a modest variation was inferred for pressure (Figures 5A–D), isotropic (Figures 6A–D) and anisotropic (Figures 7A–D) energy densities, illustrated as normalized histograms. The corresponding ECDF exhibited 3.8%–15.6% (Figures 5E–H), 1.6%–5.6% (Figures 6E–H), and 0.8%–7.9% (Figures 7E–H) variations, respectively, for the above load cases. For the applied torque of 5 Nm, the range of motion (ROM) differed moderately between the element types with a variation of 2.7%, 8.2%, 9.1%, and 9.9% for flexion, extension, lateral bending, and axial rotation, respectively.

Numerical simulation results (i.e., load-displacement curves) from FE models for non-, moderately, and severely degenerated spinal segments (Supplementary Figure S1) using HE and TE types were compared in terms of stability. All simulations converged and yielded physically meaningful results except for severely degenerated instances, where the HE approach failed in flexion and generally yielded high computational times (in extension in particular).

### 3.3 Calibration of material constants for non-degenerated IVDs

**FIGURE 8:**
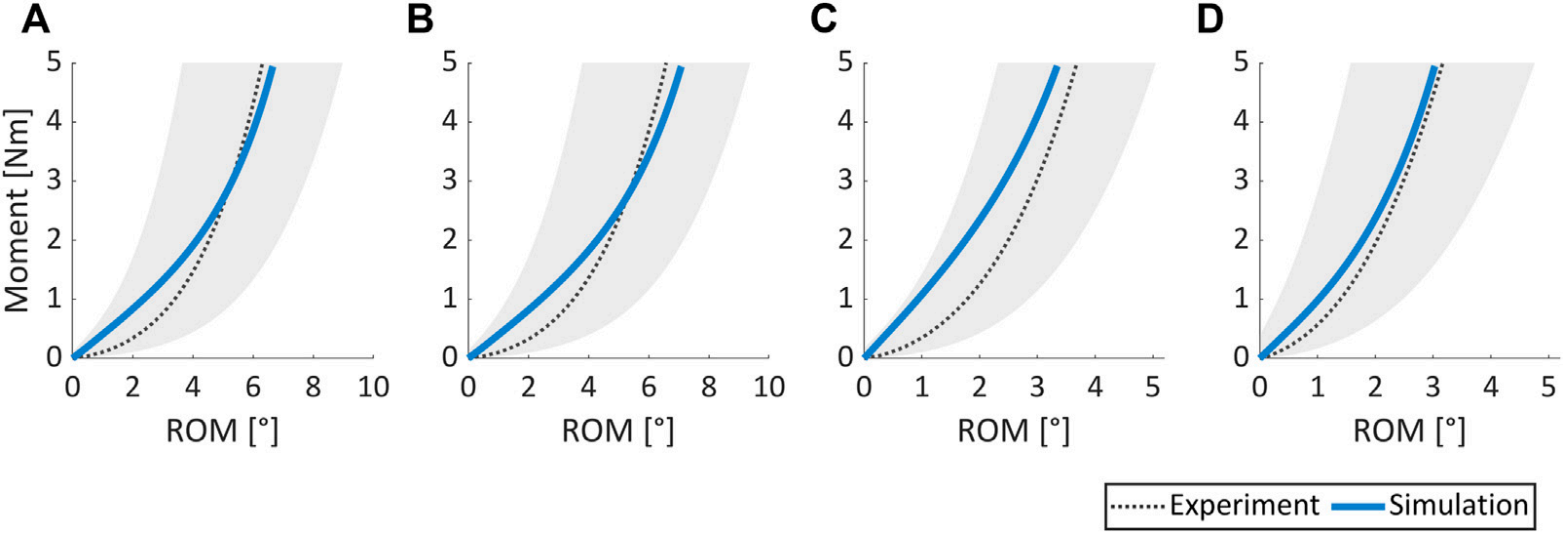
Comparison of experimental results vs. numerically simulated responses for **(A)** flexion, **(B)** extension, **(C)** lateral bending, and **(D)** axial rotation load cases. The shaded grey area covers the range of mean experimental ROM (dotted black line) ± one standard deviation. Experimental data were obtained from 31 non-degenerated spine segments (Widmer et al., 2020).

The optimal constitutive model parameter set ***p***_opt_ for the non-degenerated AF is listed in Table 3 and was deduced following the termination of the optimization algorithm (Figure 3). Figure 8 depicts the good agreement between the numerically simulated and the corresponding experimental data for all four load cases.

**TABLE 3:** Material model parameters of the AF obtained through inverse FEM and with the experimental results published in Widmer et al. (2020).

Material	Parameter	Value
Annulus fibrosus	*c*_10_ (kPa)	0.016
	*c*_01_ (kPa)	0.001
	*a*_1_ (kPa)	1
	*a*_2_	151
	*k*_AF_ (kPa)	170

## 4 Discussion

The goal of this work is to develop and validate state-of-the-art spinal FE models (developed from CT scan images) using a hyperelastic formulation for the IVDs and employing TE elements. To this end, continuum material models were used for the NP and the AF in FE models employing an adaptive refined time-stepping approach, in contrast to the traditional approaches (Freed et al., 2005; Graff, 2012; Wu et al., 2020). Furthermore, numerical simulations were performed and verified for their stability and accuracy for flexion, extension, lateral bending, and axial rotation load cases on FE models of pristine as well as moderately and severely degenerated spinal segments. Finally, a set of material constants meant to describe the average behavior of non-degenerated AF tissue was found with an optimization approach.

### 4.1 Time step estimation for explicit FEM

In a variety of problems in non-linear mechanics, Δ*t*_cric_ is often prescribed as a small enough constant to ensure the stability and accuracy of the numerical solutions. However, in the presence of material, geometrical, and contact non-linearities, much smaller time steps are generally utilized which also increases the involved computational effort. Though in the current study the latter aspects are not involved, large deformations can be expected due to material non-linearities including influences from anisotropy. This can greatly impact Δ*t*_cric_ owing to the state of deformation and stress. In this context, theoretically estimated Δ*t*_cric_ (Eq. 10) can be employed as an alternative to choosing small but arbitrary time steps. However, such an approach involves a computationally expensive iterative optimization. To this end, the approximation method presented in this work (Eq. 11) provides an excellent alternative, in that the obtained results differ only modestly from the theoretical estimates, i.e., < 10% and ≈10° in the magnitude of wave speed and the corresponding direction of propagation, respectively. Noteworthy, these differences were estimated considering all four load cases, thus involving over 2 × 10^5^ instances of {**F**, ***σ***}^e^ (Supplementary Table S1), which is a reasonable sample size.

### 4.2 Spinal model predictions for HE and TE

Only moderate differences were observed between numerical simulation results obtained with HE and TE, although the former offers increased accuracy over the latter in general (Hughes, 2012). Herein, ROM, isotropic and anisotropic energy densities differed modestly between both element types for all load cases, while for mechanical pressure moderate differences were observed (in excess of 10% for axial rotation).

Numerical simulations on non-degenerated, moderately, and severely degenerated spinal segments reveal that TE-based FE models were stable and computationally efficient in all three scenarios for all four load cases. In contrast, HE-based FE models exhibited stability only for non-degenerated and moderately degenerated scenarios involving relatively smooth IVD geometries and failed in the case of a severely degenerated IVD with a highly skewed geometry. These results reinforce the superiority of TE elements for simulations involving complex geometrical shapes in line with Schneider et al. (2019) while maintaining a homogeneous element size. Although similar results can be expected from HE types with refined and inhomogeneous meshes, the generation of these can be labor-intensive and computationally expensive.

In light of the above results involving commonly encountered load cases and various degenerative states of IVD, it is proposed that the presented TE-based FE models with component-specific models and a revised explicit time step offer a robust and computationally efficient alternative to studying the mechanics of the spine in general.

### 4.3 Calibration of material constants for non-degenerated IVDs

The AF exhibits an inhomogeneous microstructure with varying collagen directions within the lamellae. Moreover, these directions along with the amount of water in the AF are also reported to be influenced by its degenerative states. For instance, collagen fiber bundles are less organized in severely degenerated IVDs. However, in this work, the AF was represented as a micro-structurally homogeneous hyperelastic material using a 3D continuum formulation with two constant preferential directions. While this is in line with many state-of-the-art approaches [e.g., Eberlein et al. (2001); Schmidt et al. (2006; 2007a); Jaramillo et al. (2015); Ayturk et al. (2010; 2012)], noteworthy, it is more appropriate to associate such a characterization of the IVD tissue with non-degenerated states. To this end, the corresponding material parameters of the IVD were estimated using the experimental data obtained from spinal segments in a non-degenerated state. Herein, mean curves were utilized for the optimization-algorithm-driven inverse FEM, for simplicity.

Furthermore, many previous studies have utilized uni-axial experimental data for calibrating material models. However, in view of the complex IVD microstructure, model calibration-based multi-axial experimental data is highly desirable for it not only enhances the predictive abilities for generic load cases but also towards highly valued applications in clinical and subject-specific studies (Widmer, 2020; Pickering et al., 2021; Fasser et al., 2022). Therefore, in this study mean experimental data from flexion, extension, lateral bending, and axial rotation loads were employed to appropriately estimate IVD material parameters utilizing a state-of-the-art inverse FEM approach employing explicit time stepping method and driven by an optimization algorithm. Noteworthy, in the case of degenerated IVDs, the anisotropy component of SEDF in Eq. 7 can be suitably altered by employing the generalized structural tensor approach (Gasser et al., 2006; Holzapfel et al., 2015) to represent various degrees of local anisotropy.

### 4.4 Limitations

Intervertebral disc degeneration is reported to cause irreversible morphological changes such as the appearance of inhomogeneous tears and delaminations, increased disorganization in the AF microstructure (Urban and Roberts, 2003; Adams and Roughley, 2006), biochemical changes such as a decrease in water content, stiffening of the AF (Ebara et al., 1996), and geometrical changes such as an irregular but substantial reduction in height (Frobin et al., 1997); see Urban and Roberts (2003); Adams and Roughley (2006) for more details. Addressing the morphological and biochemical aspects mandates a rigorous mathematical framework involving inelastic, time-dependent, and multi-phasic effects (e.g., Cegoñino et al. (2014); Yang and O’Connell (2019)) and is beyond the scope of the current work. In this study, readily available geometry details extracted from CT scans were considered and the performance of the corresponding spinal segment FE models was explored in the realm of hyperelasticity. Furthermore, it is noted that TE-based FE discretization employed here is known to exhibit overly stiff behavior in pure displacement formulation when Poisson’s ratio approaches 0.5. The use of higher-order elements with reduced integration methods and mixed element formulations can reduce volumetric locking and improve the accuracy of numerical simulations (Joldes et al., 2009), the exploration of which is beyond the scope of this contribution.

## 5 Summary and conclusion

In this work, a continuum hyperelastic anisotropic material model was utilized to represent the AF component of the IVD. To facilitate the computational efficiency of explicit time-stepping method for spinal segment FE models, a novel approach for adaptive time step approximation was used and its proximity to theoretical estimates was evaluated. Furthermore, the effectiveness of TE-based FE models over HE-based ones was verified for various load cases when dealing with complex shapes of degenerated IVDs. Finally, a material parameter set was determined using inverse FEM and experimental data from flexion, extension, lateral bending, and axial rotation of non-degenerated human cadaveric spinal segments. Integrating the proposed approach of time step estimation with appropriately formulated TEs enables time-efficient modeling of even highly degenerated human IVD anatomies. Such anatomies often present severe challenges to automated finite element meshing strategies and/or result in suboptimal computational efficiencies to severely limit the clinical translation of patient-specific FE modeling approaches. The proposed work offers a path forward to overcoming these obstacles, without compromising on numerical accuracy. The subroutine developed in this work and used for the description of the AF material model is incorporated into Altair Radioss and will be released in the next official release in 2023.

## Data Availability

The raw data supporting the conclusion of this article will be made available by the authors, without undue reservation.

## Footnotes

1 This property exploits the identical nature of collagen fibers along ***M***_1_ and ***M***_2_.

2 For incompressible materials, ***m*** ⋅ ***n*** = 0 and hence Eqs 9, 10 must be adjusted accordingly.

3 One order of magnitude smaller than in Graff (2012), Freed et al. (2005), Wu et al. (2020).
