# Erratum: A novel approach for tetrahedral-element-based finite element simulations of anisotropic hyperelastic intervertebral disc behavior

**DOI:** 10.3389/fbioe.2023.1170903

**Published:** 2023-03-02

**Authors:** Frontiers Production Office

**Affiliations:** Frontiers Media SA, Lausanne, Switzerland

**Keywords:** intervertebral discs, spinal segments, microstructural modeling, FE-based models, explicit time-stepping, inverse FE method, tetrahedral elements

An omission to the **Funding** section of the original article was made in error. The following sentence has been added: “Open access funding was provided by ETH Zurich.”

The publisher apologizes for this mistake. The original version of this article has been updated.

