# Supplementary material for: A novel approach for tetrahedral-element-based finite element simulations of anisotropic hyperelastic intervertebral disc behavior
Source: Front Bioeng Biotechnol. 2022 Dec 13;10:1034441. doi: 10.3389/fbioe.2022.1034441 (PMC9792499; doi:10.3389/fbioe.2022.1034441)
Supplement: Supplementary file 1 [file DataSheet1.PDF]

# *Supplementary Material*

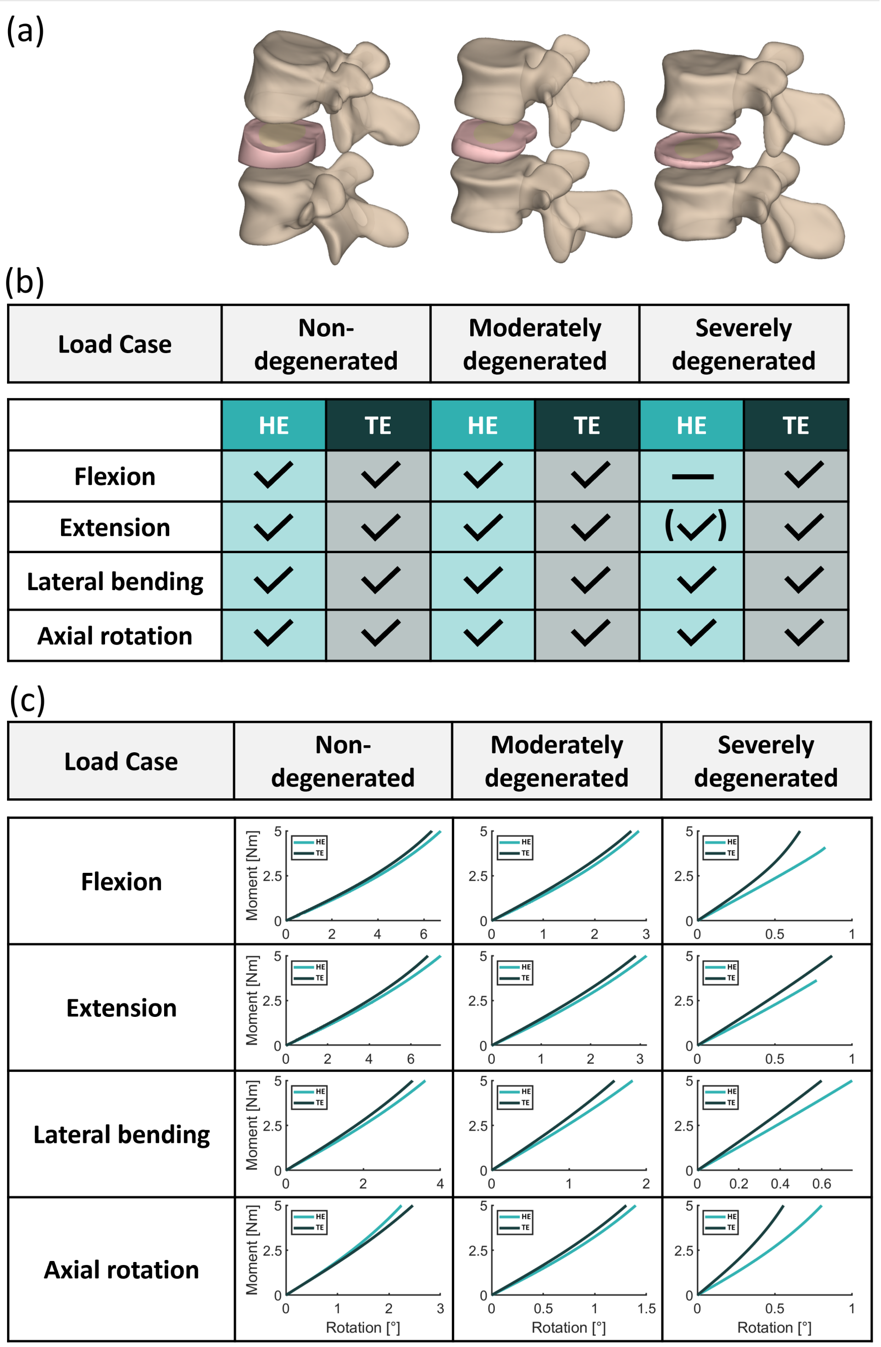


**Figure S1.** (a) 3D models with varying degrees of disc degeneration: non-degenerated (L4-L5), moderately degenerated (L1-L2), and severely degenerated (L2-L3) spine segments. The anatomical features of the vertebral bone structure were obtained from CT scans. (b) Stability of numerical simulations for all four load cases and three FE model instances using HE and TE types. The check mark signifies the obtainment of a stable simulation result, the parentheses imply an excessively low time step, and a failed simulation is indicated with a dash. (c) Plots depicting applied moment vs estimated rotations for 4 load cases applied to 3 FE geometries (total 12 cases). In general, results indicate a softer material response of HE-based FE model compared to TE-based ones, with the most noticeable differences in the predicted response observed during axial rotation.

| Degeneration | Tissue type | # elements | # nodes | Element type | Element size [mm] |
|---|---|---|---|---|---|
| Intervertebral disc mesh refinement | | | | | |
| None | NP | 9602 | 1971 | Tetrahedral | 2.20 |
| | AF | 13797 | 3043 | Tetrahedral | 2.20 |
| None | NP | 17623 | 3486 | Tetrahedral | 1.80 |
| | AF | 25333 | 5304 | Tetrahedral | 1.80 |
| None | NP | 38930 | 7388 | Tetrahedral | 1.40 |
| | AF | 54011 | 10716 | Tetrahedral | 1.40 |
| None | NP | 105978 | 19416 | Tetrahedral | 1.00 |
| | AF | 149119 | 28184 | Tetrahedral | 1.00 |
| Lumbar spine segment models in various degeneration stages | | | | | |
| None | NP | 38930 | 7388 | Tetrahedral | 1.40 |
| | AF | 54011 | 10716 | Tetrahedral | 1.40 |
| | NP | 10516 | 12024 | Hexahedral | - |
| | AF | 5940 | 7560 | Hexahedral | - |
| | Cranial vertebra | 19440 | 9720 | Shell, TRIA | 1.75 |
| | Caudal vertebra | 11250 | 5625 | Shell, TRIA | 1.75 |
| Moderate | NP | 23488 | 4640 | Tetrahedral | 1.40 |
| | AF | 38324 | 7810 | Tetrahedral | 1.40 |
| | NP | 10516 | 12024 | Hexahedral | - |
| | AF | 5940 | 7560 | Hexahedral | - |
| | Cranial vertebra | 16200 | 8100 | Shell, TRIA | 1.75 |
| | Caudal vertebra | 15000 | 7500 | Shell, TRIA | 1.75 |
| Severe | NP | 12110 | 2599 | Tetrahedral | 1.40 |
| | AF | 18978 | 4275 | Tetrahedral | 1.40 |
| | NP | 10516 | 12024 | Hexahedral | - |
| | AF | 5940 | 7560 | Hexahedral | - |
| | Cranial vertebra | 15000 | 7500 | Shell, TRIA | 1.75 |
| | Caudal vertebra | 15000 | 7500 | Shell, TRIA | 1.75 |

**Table S1.** Summary of the element types and their quantity used in the simulations with Radioss software. The listed element sizes are average values for the specified component mesh.

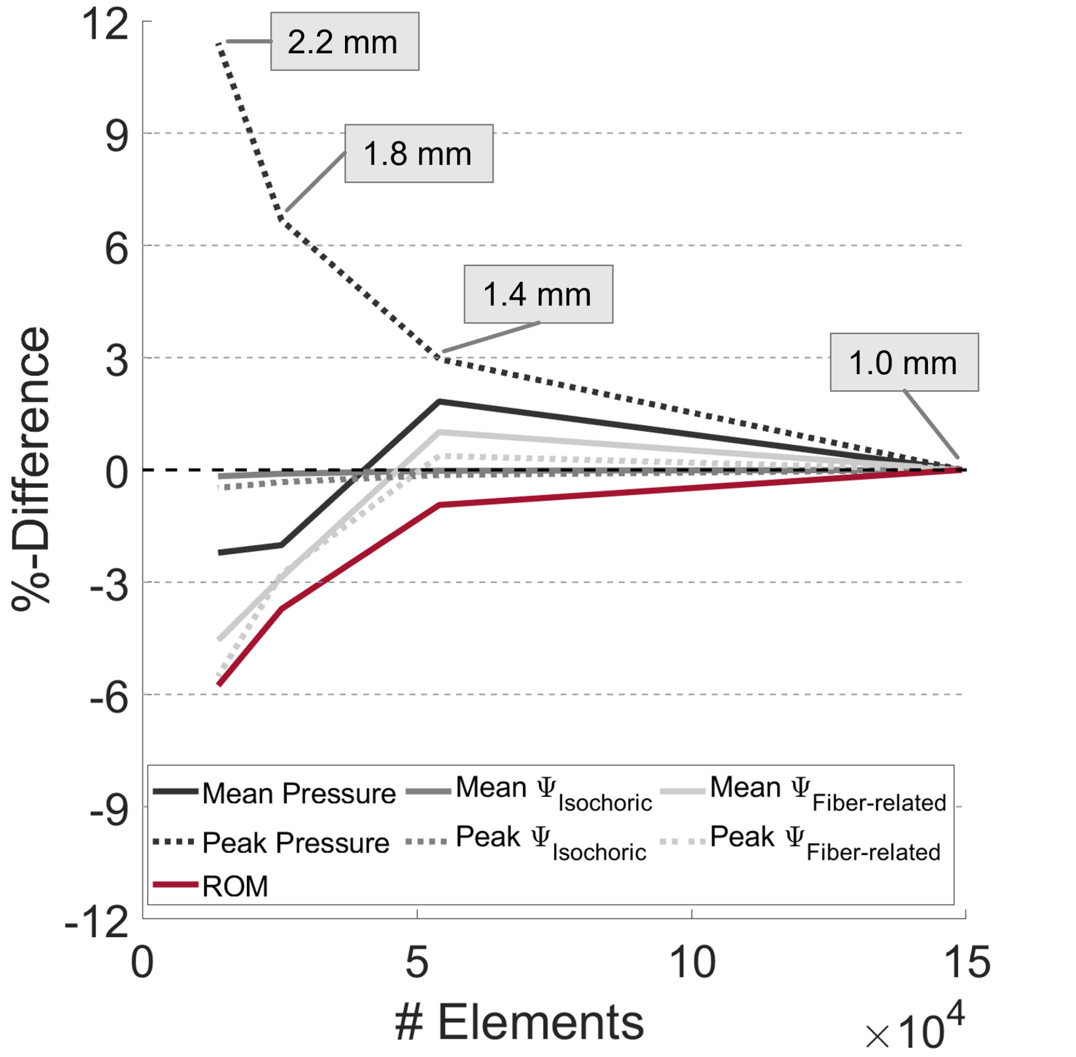


**Figure S2.** Influence of tetrahedral mesh size on relevant simulation output parameters (for the non-degenerated spinal segment loaded in flexion). The plot depicts the percentage difference from the mesh with the highest element density (i.e. average element size of 1.0 mm; Table. S1). These results indicate that a mesh with ≈54'000 elements (mean element size of 1.4 mm) differs from a refined mesh with ≈150'000 elements by less than 3% and hence is deemed computationally appropriate for FE analyses.
